# Proteomic dataset of wolframin-deficient mouse heart and skeletal muscles

**DOI:** 10.1016/j.dib.2018.10.015

**Published:** 2018-10-12

**Authors:** Margus Eimre, Sergo Kasvandik, Marilin Ivask, Sulev Kõks

**Affiliations:** a Chair of Pathological Physiology, Institute of Bio- and Translational Medicine, University of Tartu, Ravila 19, 50411 Tartu, Estonia; b Proteomics Core Facility, Institute of Technology, University of Tartu, Nooruse 1, 50411 Tartu, Estonia

**Keywords:** Wolfram syndrome, Wfs1, Myocardium, skeletal muscle, Animal proteomics

## Abstract

The data presented in this article are related to the research article entitled "Increased Mitochondrial Protein Levels and Bioenergetics in the *musculus rectus femoris* of Wfs1-Deficient mice" (Eimre et al., accepted for publication). This dataset reports the analysis of Wfs1-deficient mouse heart, *musculus soleus*, and white part of *musculus rectus femoris* by liquid chromatography/tandem mass spectrometry. Label-free quantitative analysis of the mass spectrometry data identified 4056 proteins, with 114, 212, and 1290 proteins differentially expressed (*t*-test; *p* < 0.05) in the heart, *m*. *soleus*, and *m. rectus femoris*, respectively, between the Wfs1-deficient and wild-type groups. Eight proteins were found to be differentially expressed in all mentioned muscles, with 1 protein differently expressed in oxidative (*m. soleus* and heart) and 88 in skeletal muscles. This dataset supports the cited study and can be used to extend additional analyses. Data are available via ProteomeXchange with identifier PXD011019.

**Specifications table**

**Table:** 

Subject area	*Biology*
More specific subject area	*Proteomics, pathophysiology*
Type of data	*Figure, tables*
How data was acquired	*LC–MS/MS was performed on anQ Exactive Plus tandem mass spectrometercoupled to an Ultimate 3000 RSLCnano system*
Data format	*Rawandanalyzed*
Experimental factors	*WFS1deficiency*
Experimental features	*Proteins of WFS1 deficient mouse muscles were precipitated, resuspended and analyzed by label free LC-MS/MS. Label-free quantitation on the mass spectrometer data files was performed with MaxQuant*
Data source location	*Tartu, Estonia*
Data accessibility	*The mass spectrometry proteomics data have been deposited to the ProteomeXchange Consortium via the PRIDE partner repository with the dataset identifierPXD011019*.

**Value of the data**

• This is the first proteomic dataset of Wfs1 deficient muscles.
• The data may be a valuable starting point for studying the direct and indirect mechanisms of Wfs1 deficiency on mouse muscles.
• These data and further experiments based on these data may provide valuable information for understanding the mechanisms of Wolfram syndrome and type 1 diabetes.

## 1 Data

**Fig. 1:**
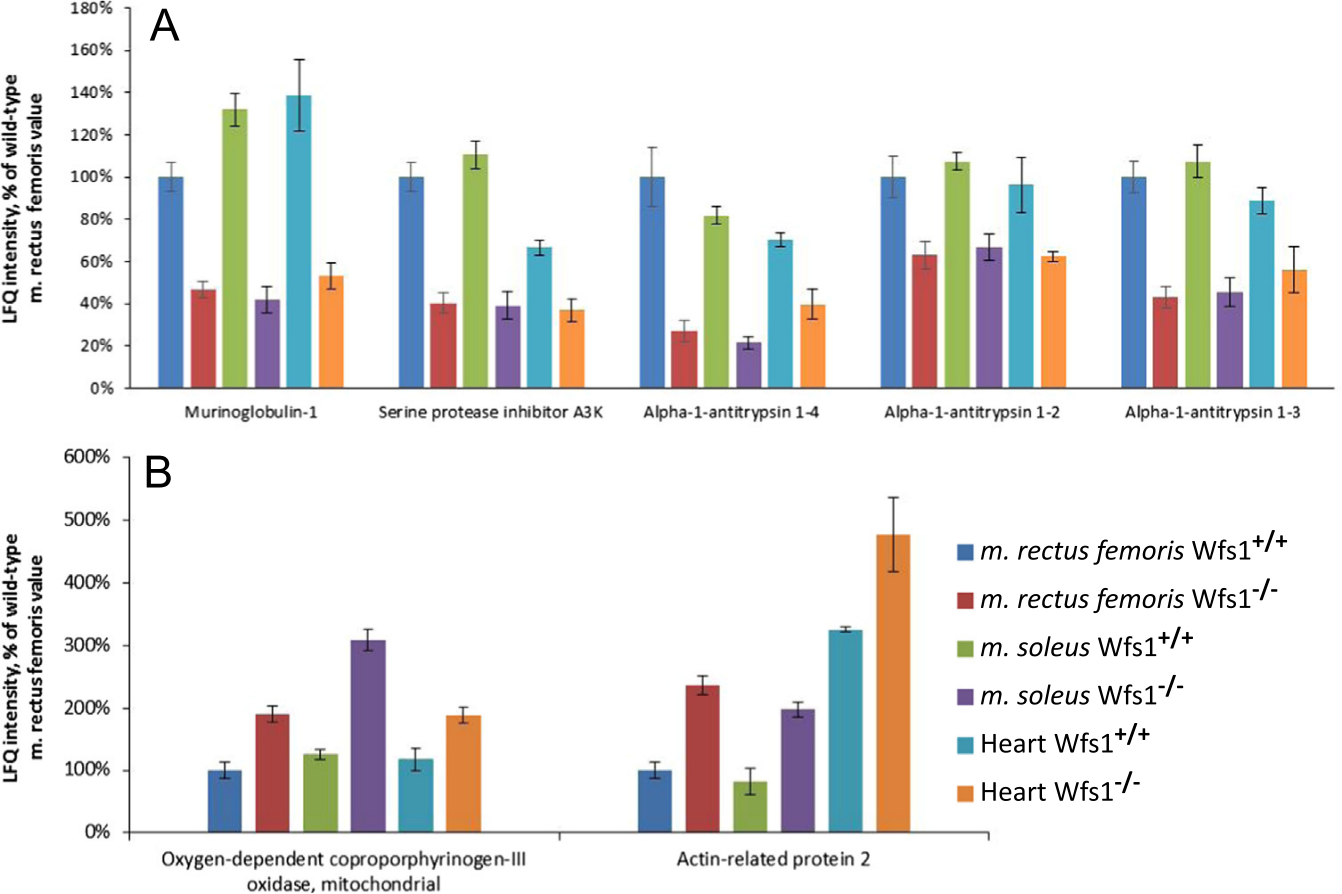
Proteins showing higher (A) and lower (B) expression proteins in all studied Wfs1-deficient muscles. Data are presented as mean ± SEM, *p*< 0.5 compared to wild-type.

Proteins found to be differentially expressed in all studied Wfs1-deficient muscles are presented in Fig. 1 and at the beginning of Table S4. The level of Bcl2-associated agonist of the cell death protein was decreased in both oxidative muscles (Table S4). Protein expression in wolframin-deficient skeletal muscle was compared to that in wild-type, which showed that the expression of 35 proteins was decreased, while 61 proteins were increased (Table 1). Data of differentially expressed proteins in the woframin-deficient heart are in Table S1, wolframin-deficient *musculus soleus* in Table S2, and woframin-deficient *musculus rectus femoris* in Table S3. Label-free quantitative (LFQ) intensities and other information for all proteins identified by liquid chromatography (LC)/tandem mass spectrometry (MS/MS) analysis of muscles are in Tables S4 and S5 (P3; P5; P6; P8; P17; P21; P23; P25: *m. rectus femoris* in wild-type mice; P1; P19; P22; P24; P26: *m. rectus femoris* in Wfs1-deficient mice; P4; P7; P18: *m. soleus* in wild-type mice; P2; P20: *m. soleus* of Wfs1-deficient mice; P9; P11; P12; P14: heart in wild-type mice; P10; P13; P15; P16: heart in Wfs1-deficient mice). All peptides identified and quantified are shown in Table S6.

## 2 Experimental design, materials and methods

### 2.1 Animals and proteomics sample preparation

The heart, *m. soleus*, and white glycolytic part of the *m. rectus femoris* from 9–12-month-old Wfs1 KO male mice and their wild-type littermates were used for LC/MS/MS analysis. The animals were housed under standard laboratory conditions on a 12-h light-dark cycle (lights on at 07:00) with free access to food and water. Experiments in this study were performed in accordance with the European Parliament Directive 2010/63/EU and permit (No. 86, May 4, 2016) from the Estonian National Board of Animal Experiments. Muscle tissues were homogenized by sonication (Bandelin Sonopuls HD 2200, Sigma-Aldrich, St. Louis, MO, USA) on ice. Proteins in the homogenates were precipitated, suspended, and digested with trypsin. The obtained peptides were desalted and reconstituted in 0.5% trifluoroacetic acid [1].

### 2.2 Proteomics data analysis

LC/MS/MS analysis was performed using an Ultimate 3000 RSLCnano system (Dionex, Sunnyvale, CA, USA) and Q Exactive Plus (Thermo Fisher Scientific, Waltham, MA, USA) tandem mass spectrometer.

Mass spectrometric raw data were processed using the MaxQuant 1.5.3.17 software package [2]. LFQ was conducted using the MaxQuant LFQ algorithm [3]. A search was performed against the UniProt (www.uniprot.org) *Mus musculus* reference proteome database (downloaded on November 11, 2015; 57,320 entries). The peptide-spectrum match and protein false discovery rate was kept below 1% using a target-decoy approach [1]. Statistical analysis of LFQ intensities of proteins was performed by Student׳s *t*-test. Data are given as the mean ± standard error of the mean. A value of *p* < 0.05 was considered statistically significant.
