# Supplementary material for: Proteomic dataset of wolframin-deficient mouse heart and skeletal muscles
Source: Data Brief. 2018 Oct 12;21:616–9. doi: 10.1016/j.dib.2018.10.015 (PMC6202784; doi:10.1016/j.dib.2018.10.015)
Supplement: Supplementary file 5 — Supplementary material [file mmc5.docx]

Table 1. Differentially (p < 0.05 compared to wild-type) expressed proteins or their subunits of studied Wfs1 deficient skeletal muscles.

| **Protein names and IDs** | m. rectus femoris | | m. soleus |  |
| --- | --- | --- | --- | --- |
|  | **Wfs1^-/-^ LFQ**  **intensity, % of wild-type value** | **p** | **Wfs1^-/-^ LFQ**  **intensity, % of wild-type value** | **p** |
| Annexin A11; Annexin; P97384; D3Z7U0 | 24% | 0.0000002 | 24% | 0.007 |
| Serine protease inhibitor A3K; P07759;  D3Z450 | 40% | 0.0001 | 35% | 0.005 |
| Murinoglobulin-1; P28665 | 47% | 0.0001 | 32% | 0.004 |
| Cysteine and histidine-rich domain-containing protein 1; Q9D1P4 | 46% | 0.0001 | 42% | 0.01 |
| Alpha-1-antitrypsin 1-3; Q00896 | 43% | 0.0001 | 42% | 0.01 |
| Peroxiredoxin-1; P35700; B1AXW5; B1AXW6; B1AXW4 | 62% | 0.0002 | 90% | 0.01 |
| Ubiquitin-associated domain-containing protein 1; Q8VDI7; F6UMH9; F6Z1E7 | 48% | 0.0003 | 37% | 0.03 |
| Vacuolar protein sorting-associated protein 37C; Q8R105 | 12% | 0.0003 | 0% | 0.003 |
| Vacuolar protein sorting-associated protein 28 homolog; Q9D1C8 | 37% | 0.0003 | 52% | 0.03 |
| Voltage-dependent L-type calcium channel subunit beta-1; A2A542; A2A545; Q8R3Z5;  A2A543; J3QK20; Q8R0S4 | 51% | 0.0004 | 53% | 0.02 |
| Voltage-dependent L-type calcium channel subunit beta-3; P54285; D3Z3Z3 | 26% | 0.0008 | 0% | 0.003 |
| Cullin-associated NEDD8-dissociated protein 2; Q6ZQ73 | 28% | 0.0009 | 30% | 0.02 |
| Protein unc-45 homolog B; Q8CGY6-2; Q8CGY6 | 52% | 0.0009 | 53% | 0.049 |
| Peflin; Q8BFY6 | 21% | 0.001 | 36% | 0.003 |
| Cullin-2; Q9D4H8; E0CYT5; H7BX52 | 42% | 0.002 | 37% | 0.02 |
| Exportin-2; Q9ERK4; E9Q1T9; F6ZEW4;  E9QAX7; F7D1H9 | 28% | 0.002 | 29% | 0.04 |
| Proteasome-associated protein ECM29 homolog; Q6PDI5; A2ALV7; A2ALV6; A2ALV8; A2ALW1; A2ALV9 | 35% | 0.002 | 31% | 0.02 |
| Signal transducer and activator of transcription 5B; P42232 | 48% | 0.002 | 48% | 0.047 |
| Alpha-1-antitrypsin 1-4; Q00897 | 27% | 0.002 | 26% | 0.002 |
| Kelch-like protein 31; G3X9D8; Q8BWA5 | 46% | 0.004 | 50% | 0.02 |
| Ancient ubiquitous protein 1; Q3U3K9;  P70295 | 51% | 0.004 | 0% | 0.02 |
| Chloride channel protein; chloride channel protein 1; F6QI82; Q64347; Q3TTA5;  F6QL23; F7A9J6; E9Q2N3; F7C9S1; F6X177 | 37% | 0.006 | 21% | 0.02 |
| Probable threonine--tRNA ligase 2, cytoplasmic; Q8BLY2 | 13% | 0.007 | 26% | 0.02 |
| E3 ubiquitin-protein ligase UBR4; A2AN08; F6SSP6; Z4YMA7 | 36% | 0.007 | 21% | 0.03 |
| Paxillin; F8VQ28; Q8VI36; A0A0J9YV30;  A0A0J9YV66 | 67% | 0.008 | 61% | 0.04 |
| Pantothenate kinase 4; Q80YV4; F7B6K4;  Q80YV4-3 | 50% | 0.009 | 54% | 0.04 |
| Cullin-4A; Q3TCH7; E9PXT5; F6UV36 | 53% | 0.01 | 49% | 0.045 |
| Peptidyl-prolyl cis-trans isomerase D;  Q9CR16; A0A0A6YW70 | 58% | 0.01 | 48% | 0.004 |
| Microtubule-associated protein RP/EB family member 2; D3YYK8; E9Q6X0; Q8R001; Q3TG90 | 58% | 0.01 | 34% | 0.048 |
| E3 ubiquitin-protein ligase RBX1; E3 ubiquitin-protein ligase RBX1, N-terminally processed; P62878 | 64% | 0.01 | 68% | 0.01 |
| Alpha-1-antitrypsin 1-2; P22599 | 63% | 0.02 | 62% | 0.01 |
| Dynamin-1-like protein; Q8K1M6-3 | 63% | 0.03 | 27% | 6.18E-05 |
| 5-Oxoprolinase; Q8K010; E9Q484; E9PZB8 | 76% | 0.03 | 66% | 0.045 |
| T-complex protein 1 subunit; Q3TIJ7;  P80313 | 79% | 0.04 | 77% | 0.02 |
| Ubiquitin carboxyl-terminal hydrolase 15;  Q8R5H1-5; Q8R5H1; Q8R5H1-2; Q8R5H1-3 | 67% | 0.047 | 61% | 0.007 |
| Q9DAV6; Q9D6A7; I7HJI5 | 616% | 0.0000002 | 314% | 0.03 |
| Q8VCQ8; E9QA16; S4R1T7; E9Q0M9;  F6QLP8; F6RGN9; E9Q9F3 | 1277% | 0.0000006 | 357% | 0.02 |
| Ubiquitin-like modifier-activating enzyme 5; Q8VE47; F6VRI6; D6RH76 | 289% | 0.000001 | 318% | 0.049 |
| 78-kDa glucose-regulated protein; P20029 | 409% | 0.000001 | 439% | 0.01 |
| Hemoglobin subunit alpha; Q91VB8; P01942; Q78PA4; A7M7S6; P06467 | 170% | 0.000002 | 154% | 0.02 |
| Nucleobindin-1; Q02819; H3BK79; D3Z7D7; D3Z1N1 | 202% | 0.000002 | 203% | 0.03 |
| Protein disulfide-isomerase A6; Q922R8;  Q3TML0 | 312% | 0.00001 | 325% | 0.0005 |
| Leukocyte elastase inhibitor A; Q9D154;  Z4YK03; Q5SV42 | 541% | 0.00001 | 281% | 0.044 |
| Hypoxia up-regulated protein 1; Q9JKR6;  F6TRP3; E0CYZ2 | 196% | 0.00003 | 159% | 0.02 |
| Actin-related protein 2; Q5SW83; P61161 | 236% | 0.00003 | 243% | 0.03 |
| Endoplasmin; Q3UAD6; P08113; F7C312 | 163% | 0.00003 | 156% | 0.02 |
| Phosphoenolpyruvate carboxykinase [GTP], mitochondrial; Q8BH04 | 1398% | 0.00003 | 562% | 0.0004 |
| Presequence protease, mitochondrial; Q8K411-2; Q8K411; Q8K411-3 | 207% | 0.00005 | 158% | 0.01 |
| UPF0598 protein C8orf82 homolog; Q8VE95 | 236% | 0.0001 | 126% | 0.002 |
| Mesencephalic astrocyte-derived neurotrophic factor; Q80ZP8; Q3TMX5;  Q9CXI5; F6USD5; F6T4L3; F7C1S6 | 649% | 0.0001 | 551% | 0.048 |
| Calreticulin; B2MWM9; P14211 | 162% | 0.0001 | 181% | 0.047 |
| Nucleobindin-2; Nesfatin-1; Q3UKN6; P81117; S4R2R9 | 1807% | 0.0002 | 1247% | 0.040 |
| Fascin; Q61553; A0A0G2JDU7; D3Z1X1; F7BDR1; D3YWW3 | 297% | 0.0002 | 199% | 0.02 |
| Staphylococcal nuclease domain-containing protein 1; Q78PY7; Q3TJ56;  E9Q3E9 | 426% | 0.0002 | 537% | 0.043 |
| Hexokinase-1; Hexokinase; G3UVV4; P17710; D3YYR4; D3Z365; D3Z105; B4YB29 | 594% | 0.0003 | 258% | 0.01 |
| RNA binding motif protein, X-linked-like-1; Q91VM5 | 775% | 0.0003 | 510% | 0.01 |
| Mitochondrial 10-formyltetrahydrofolate dehydrogenase; Q8K009; D3Z6B9 | #DIV/0! | 0.0004 | #DIV/0! | 0.002 |
| Protein disulfide-isomerase; P09103; E9Q8G8 | 137% | 0.0004 | 177% | 0.008 |
| 5-phosphohydroxy-L-lysine phospho-lyase;  Q8R1K4; F8WHK6 | 321% | 0.0004 | 192% | 0.005 |
| Aly/REF export factor 2; THO complex subunit 4; G3X9I4; Q9JJW6; O08583 | 416% | 0.0005 | 324% | 0.006 |
| Oxygen-dependent coproporphyrinogen-III oxidase, mitochondrial; P36552 | 190% | 0.0006 | 246% | 0.002 |
| Coronin; Coronin-1A; Q3U1N0; O89053;  G3UYK8; D3YW57; G3UX53; D3YXM2 | 316% | 0.0008 | 929% | 0.02 |
| Mannose-1-phosphate guanyltransferase beta; Q8BTZ7 | 147% | 0.0009 | 175% | 0.005 |
| 5(3)-Deoxyribonucleotidase, cytosolic type  Q9JM14; A2A9X5 | 216% | 0.0012 | 184% | 0.005 |
| Glypican-1; Secreted glypican-1; Q3U379;  Q9QZF2 | 159% | 0.0013 | 335% | 0.006 |
| Cofilin-1; P18760; F8WGL3 | 182% | 0.0015 | 177% | 0.04 |
| Translin-associated protein X; Q9QZE7 | 212% | 0.0015 | 149% | 0.02 |
| D-3-Phosphoglycerate dehydrogenase; Q61753; F6ZSB7 | 2521% | 0.0016 | 1336% | 0.01 |
| Protein disulfide-isomerase A4; P08003 | 246% | 0.0025 | 235% | 0.045 |
| Glutamine synthetase; P15105; D3YVK1;  D3Z121 | 380% | 0.0027 | 210% | 0.02 |
| Malectin; D3Z1M3; Q6ZQI3 | 234% | 0.0035 | #DIV/0! | 0.001 |
| Ankyrin-3; S4R2S8; G5E8K2; G5E8K3;  G5E8K5; S4R1S2; S4R2K9; S4R2J6; S4R165 | 172% | 0.0045 | 313% | 0.01 |
| Coatomer subunit delta; Q5XJY5 | 148% | 0.0048 | 186% | 0.02 |
| Vinculin; Q64727 | 115% | 0.0061 | 134% | 0.005 |
| Vimentin; Q5FWJ3; P20152; A0A0A6YWC8;  A2AKJ2; P03995 | 264% | 0.0067 | 295% | 0.03 |
| Elongation factor 1-beta; O70251; G3UX43; A0A087WS46; M0QWK5 | 148% | 0.0075 | 182% | 0.01 |
| A3KGU9; A3KGU7; A3KGU4 | 279% | 0.0075 | 283% | 0.01 |
| RNA-binding protein 39; Q8VH51; F7AA45;  E9Q8F0; B7ZD61 | 287% | 0.0076 | 625% | 0.03 |
| Stromal cell-derived factor 2-like protein 1;  Q9ESP1 | 1831% | 0.0081 | 1810% | 0.047 |
| Plastin-2; Q61233; D3YZ25; D3YVW8;  D3Z7D9; D3Z311 | 209% | 0.0094 | 228% | 0.03 |
| A0A087WQS0; A0A087WQM0; E9Q0S6; A0A087WQ94; A0A087WRU0; Q9DBT6;  A0A087WPC7; A0A087WR29; A0A087WP40; Q5SSZ5-2; A0A087WSC8; A0A087WS32 | 192% | 0.0113 | 768% | 0.003 |
| Protein disulfide-isomerase A3; P27773; F6Q404 | 149% | 0.0119 | 155% | 0.008 |
| Protein canopy homolog 3; Q9DAU1; Q9DAU1-2 | 234% | 0.0134 | 234% | 0.03 |
| Glutathione peroxidase 1; glutathione peroxidase; P11352; A0A0A6YVV2 | 151% | 0.0154 | 196% | 0.045 |
| Microtubule-associated protein 1B; MAP1B heavy chain; MAP1 light chain LC1; P14873 | 183% | 0.0155 | 347% | 0.03 |
| Starch-binding domain-containing protein 1; Q8C7E7; A0A0J9YUR6 | 142% | 0.0164 | 328% | 0.03 |
| Complement factor D; P03953-2; P03953 | 191% | 0.0195 | 214% | 0.02 |
| Coronin-7; Q9D2V7; G3X9L5; E9PYU1 | 201% | 0.0209 | 356% | 0.045 |
| Vacuolar protein sorting-associated protein 45; P97390 | 1881% | 0.0217 | #DIV/0! | 0.0007 |
| Serine/threonine-protein phosphatase PP1-alpha catalytic subunit; P62137 | 123% | 0.0222 | 192% | 0.02 |
| Non-POU domain-containing octamer-binding protein; Q99K48; Q99K48-2 | 227% | 0.0271 | 1452% | 0.04 |
| Aldehyde dehydrogenase family 16 member A1; D3Z0B9; Q571I9; F6RQF0 | 175% | 0.0278 | #DIV/0! | 0.001 |
| DDRGK domain-containing protein 1; Q80WW9; B0R015 | 329% | 0.0284 | 305% | 0.03 |
| Vesicle-trafficking protein SEC22b; O08547; E9Q6R3; A0A0G2JF08; D6RES2 | 150% | 0.0288 | 159% | 0.03 |
| Ras-related C3 botulinum toxin substrate 2; Q05144 | 340% | 0.0403 | #DIV/0! | 0.001 |
| Ubiquitin-fold modifier 1; P61961; H7BWZ1; D3YW97 | 138% | 0.0407 | 510% | 0.02 |
